# Supplementary figures and images for: Profusion of G-quadruplexes on both subunits of metazoan ribosomes
Source: PLoS One. 2019 Dec 13;14(12):e0226177. doi: 10.1371/journal.pone.0226177 (PMC6910669; doi:10.1371/journal.pone.0226177)

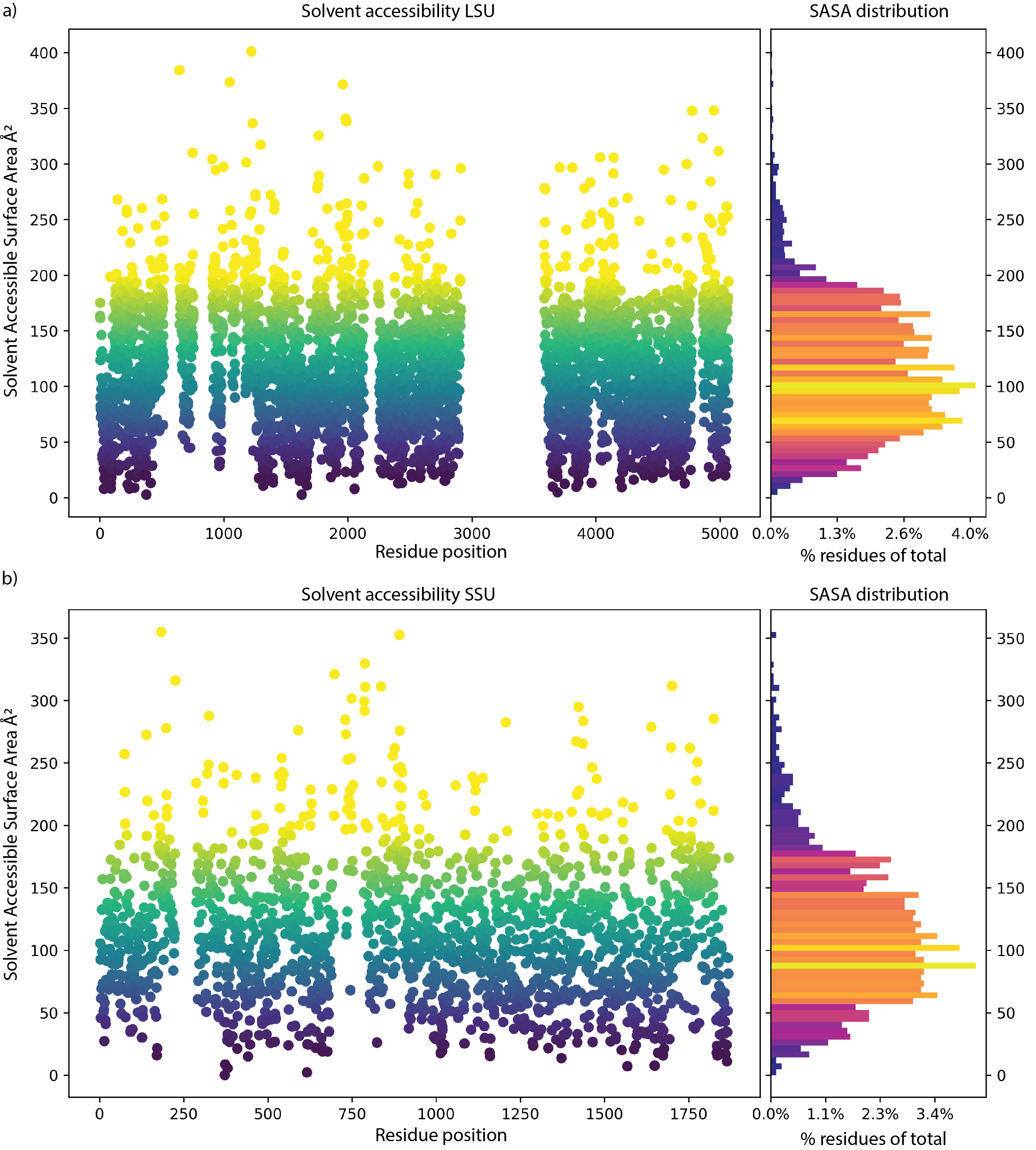

Supplement: S1 Fig — (JPG) [file pone.0226177.s001.jpg]

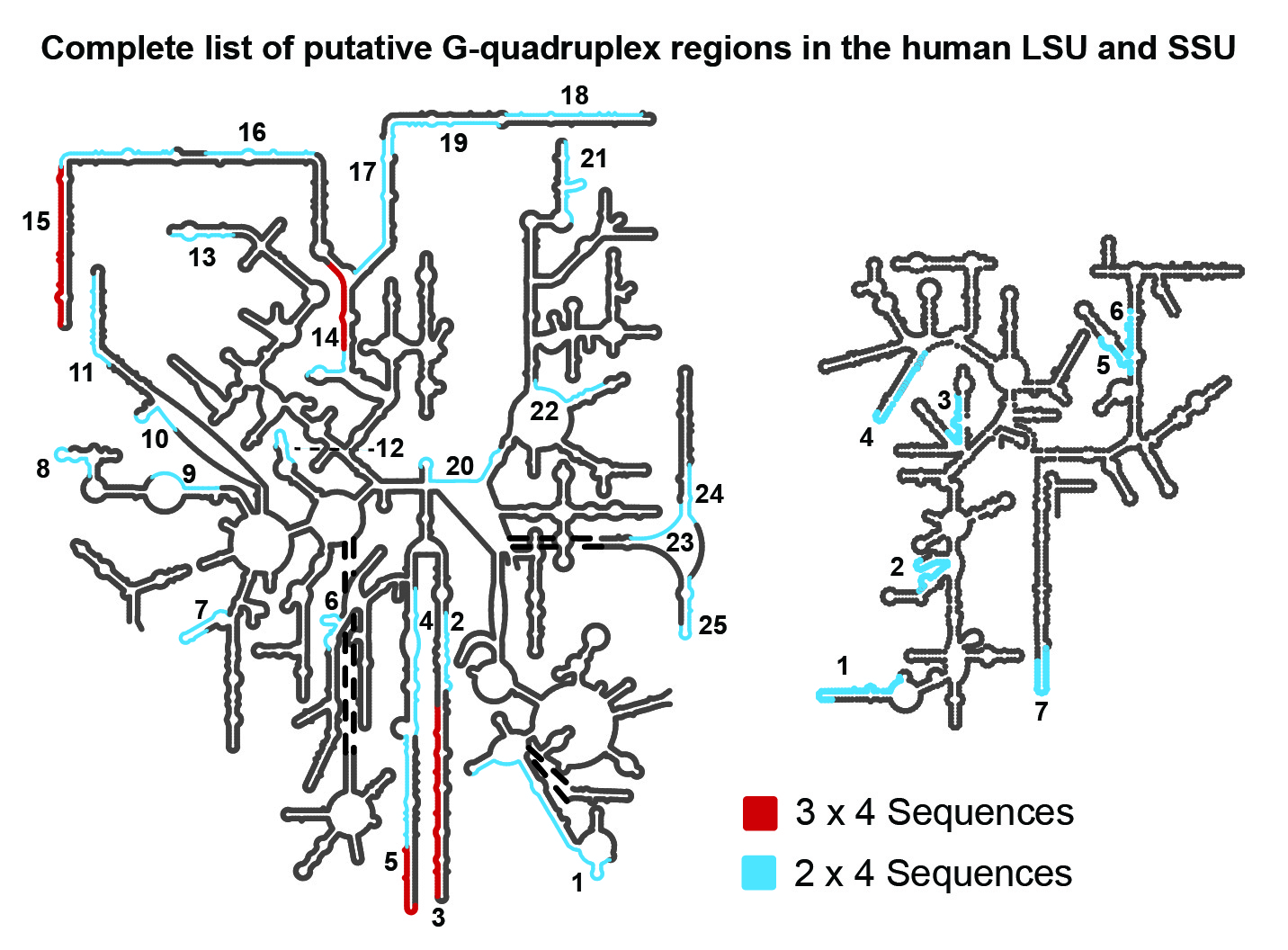

Supplement: S2 Fig — (JPG) [file pone.0226177.s002.jpg]

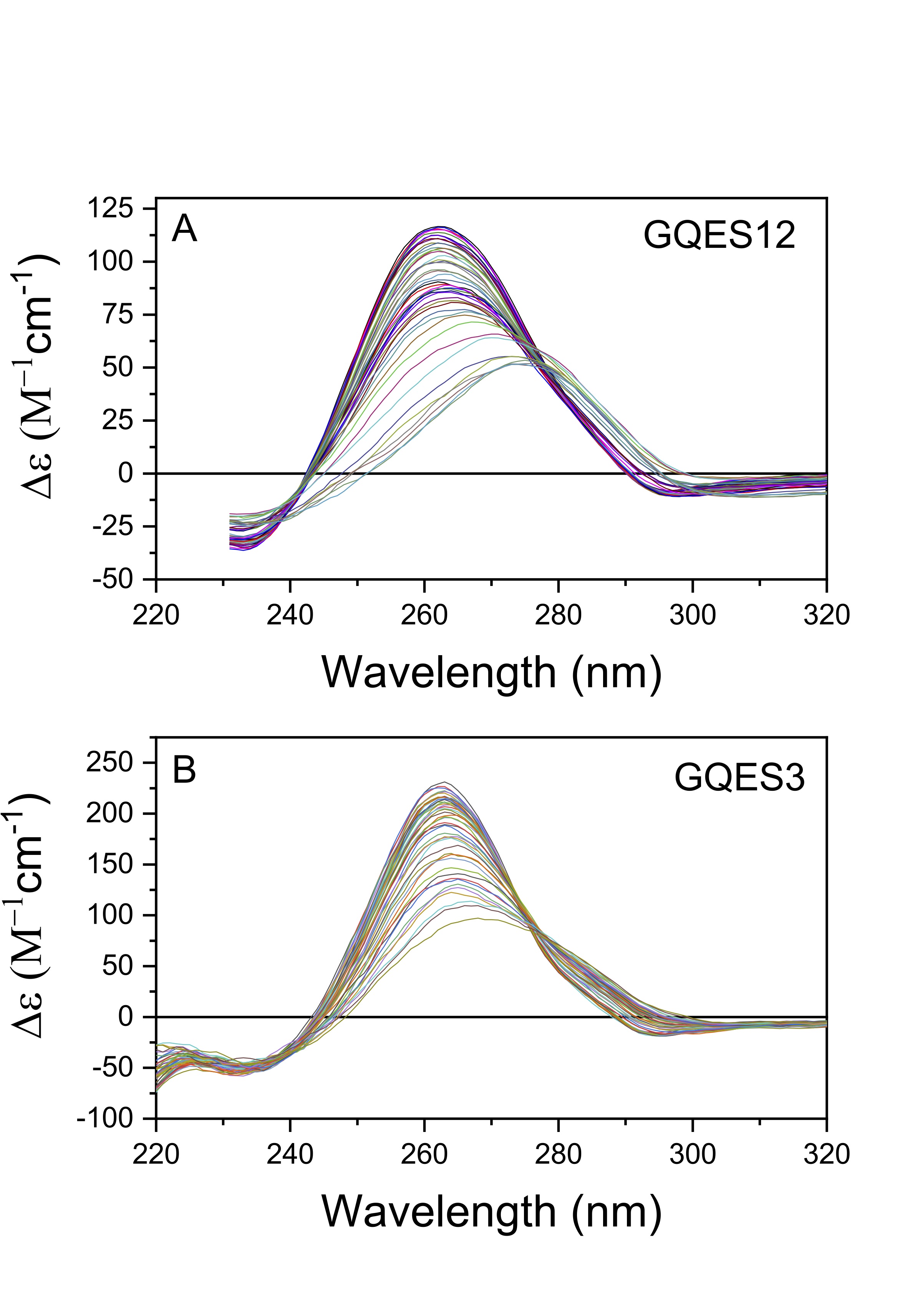

Supplement: S3 Fig — The amplitude of the CD signal near 260 nm decreases as temperature is increased. (JPG) [file pone.0226177.s003.jpg]

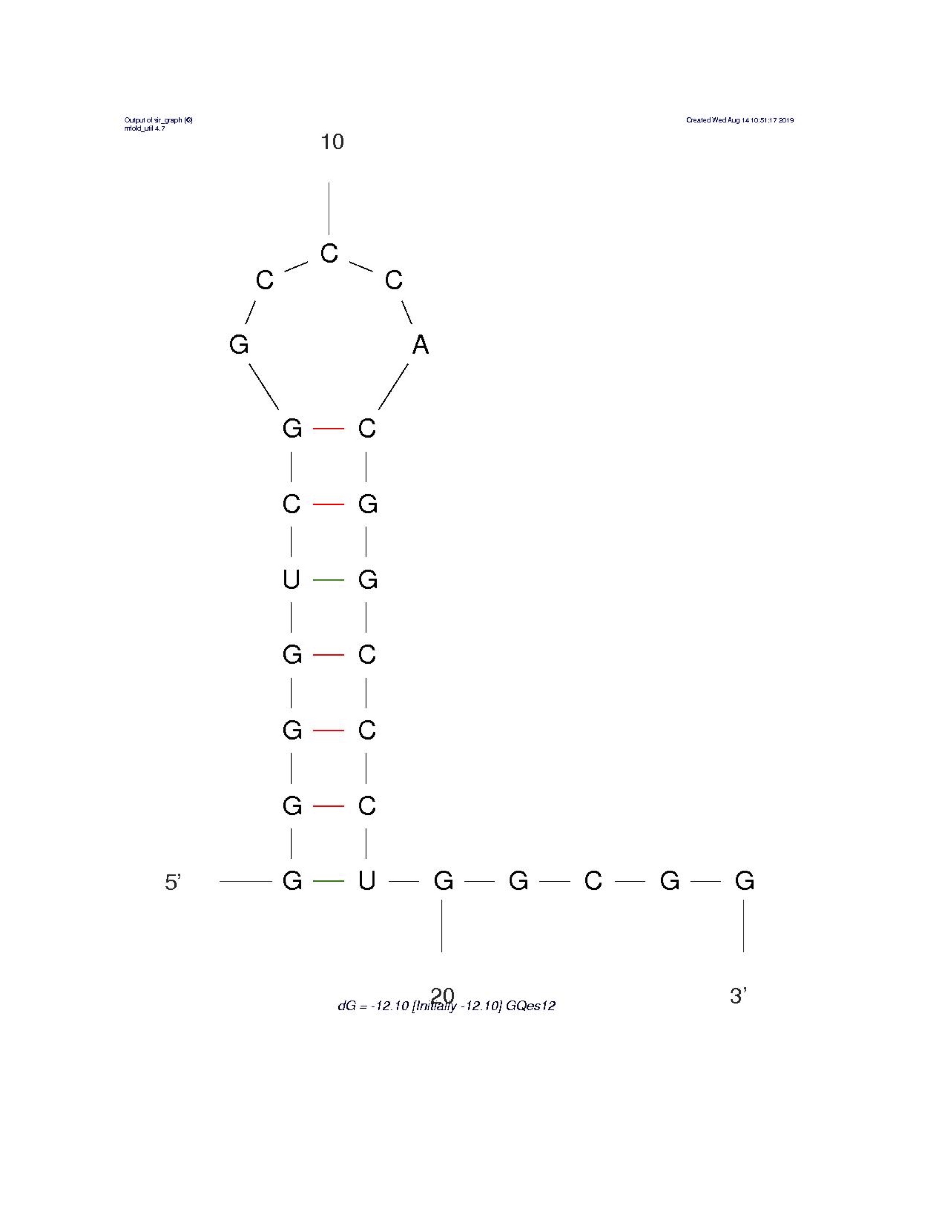

Supplement: S4 Fig — (JPG) [file pone.0226177.s004.jpg]

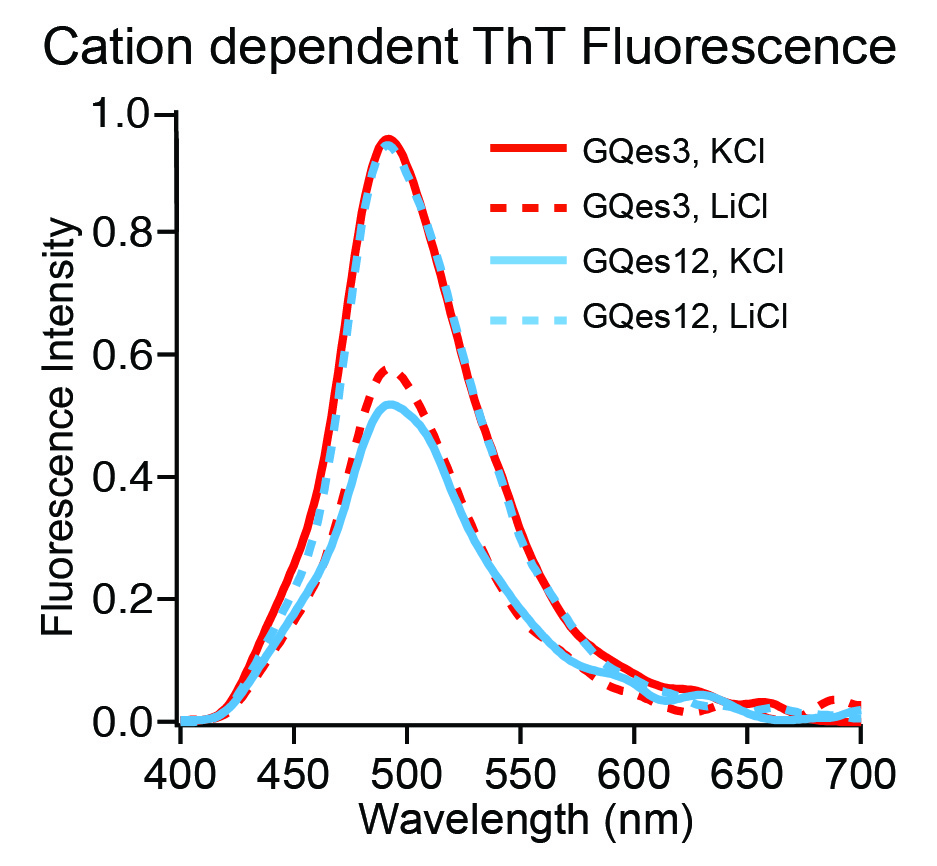

Supplement: S5 Fig — (JPG) [file pone.0226177.s005.jpg]

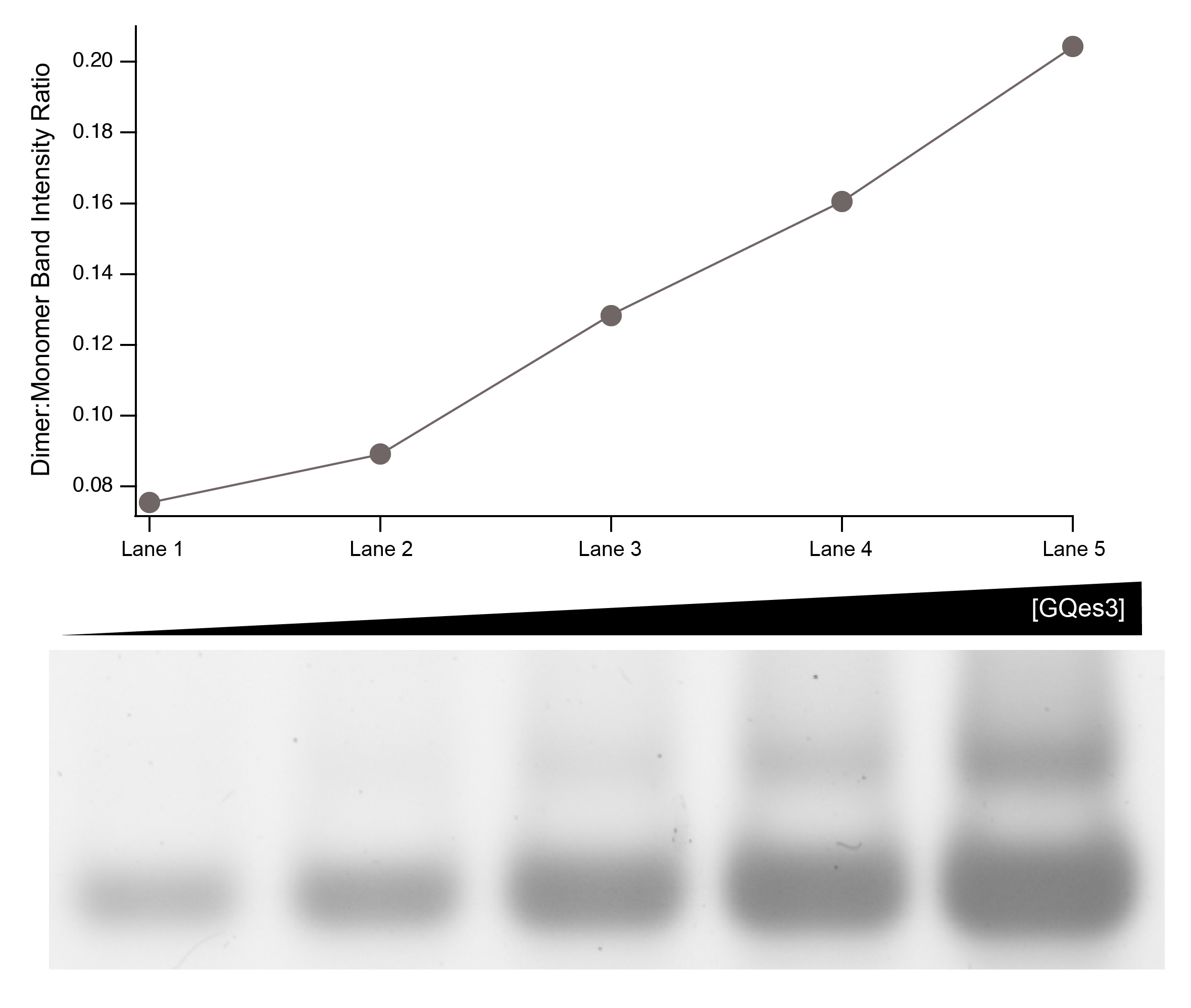

Supplement: S6 Fig — Band intensities were quantified using ImageJ and the ratio of dimer to monomer was plotted. The increase in the ratio indicates that the equilibrium is shifted from monomer to dimer upon increase in the RNA concentration. RNA was resolved on a 6% Native PAGE. (JPG) [file pone.0226177.s006.jpg]
