# Supplementary material for: Profusion of G-quadruplexes on both subunits of metazoan ribosomes
Source: PLoS One. 2019 Dec 13;14(12):e0226177. doi: 10.1371/journal.pone.0226177 (PMC6910669; doi:10.1371/journal.pone.0226177)
Supplement: S1 Table — (DOCX) [file pone.0226177.s007.docx]

**SUPPLEMENTARY MATERIALS**

**Profusion of G-Quadruplexes in both Subunits of Metazoan Ribosomes**

Santi Mestre-Fos^1,2^, Petar I. Penev^1,3^, John C. Richards^1,2^, William L. Dean^4^, Robert D. Gray^4^, Jonathan B. Chaires^4^ and Loren Dean Williams^1,2,3^†

^1^Center for the Origin of Life, Georgia Institute of Technology,

Atlanta, GA 30332-0400, USA

^2^School of Chemistry and Biochemistry, Georgia Institute of Technology,

Atlanta, GA 30332-0400, USA

^3^School of Biological Sciences, Georgia Institute of Technology,

Atlanta, GA 30332-0400, USA

^4^James Graham Brown Cancer Center, University of Louisville,

Louisville, KY 40202, USA

Running title: **G-quadruplexes in the human SSU rRNA**

† To whom correspondence may be addressed. Email: [loren.williams@chemistry.gatech.edu](mailto:loren.williams@chemistry.gatech.edu)

**
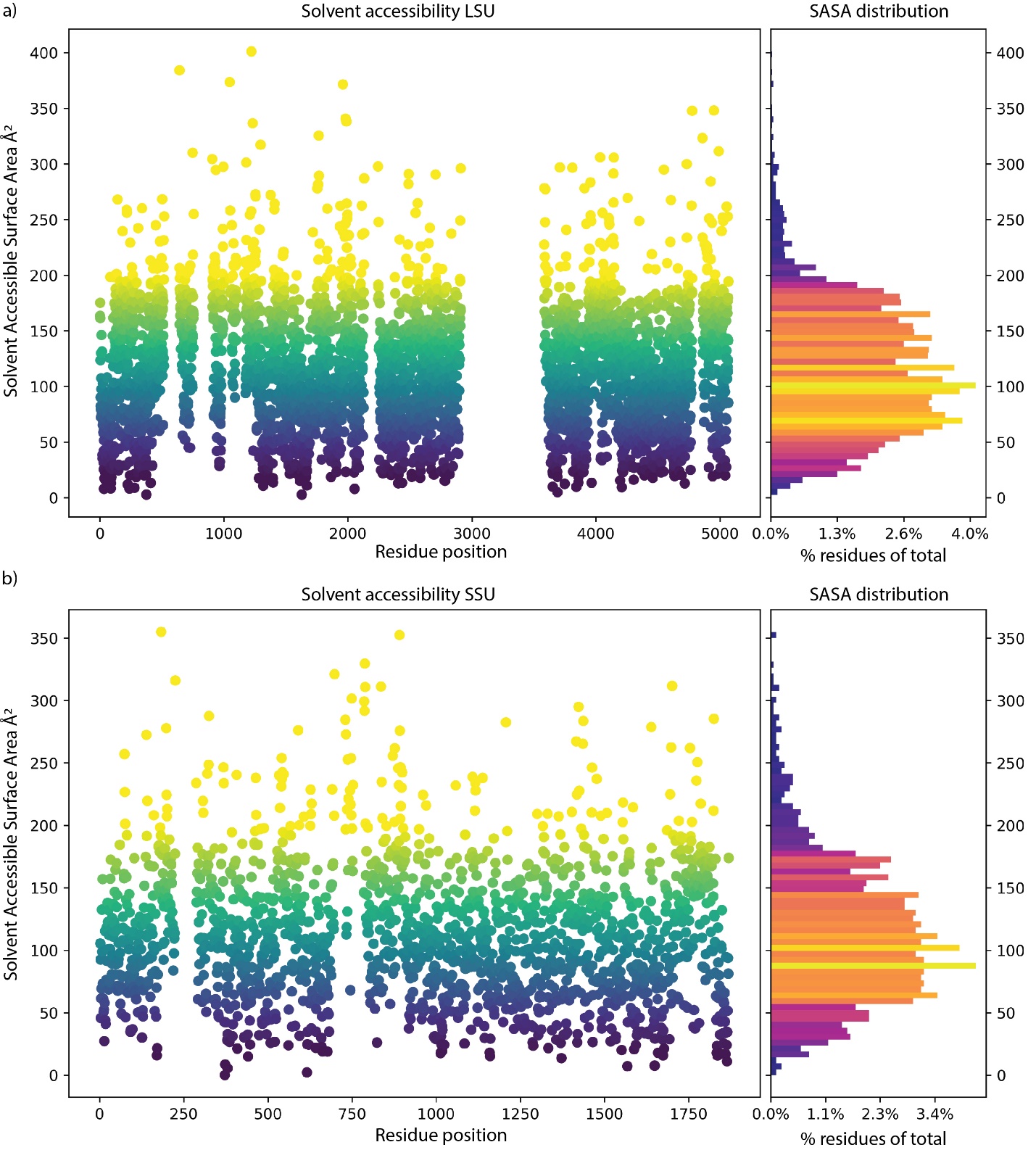
**

**Fig S1.** Distribution of solvent-accessible surface area for each residue of the a) human LSU rRNA and b) human SSU rRNA.

**
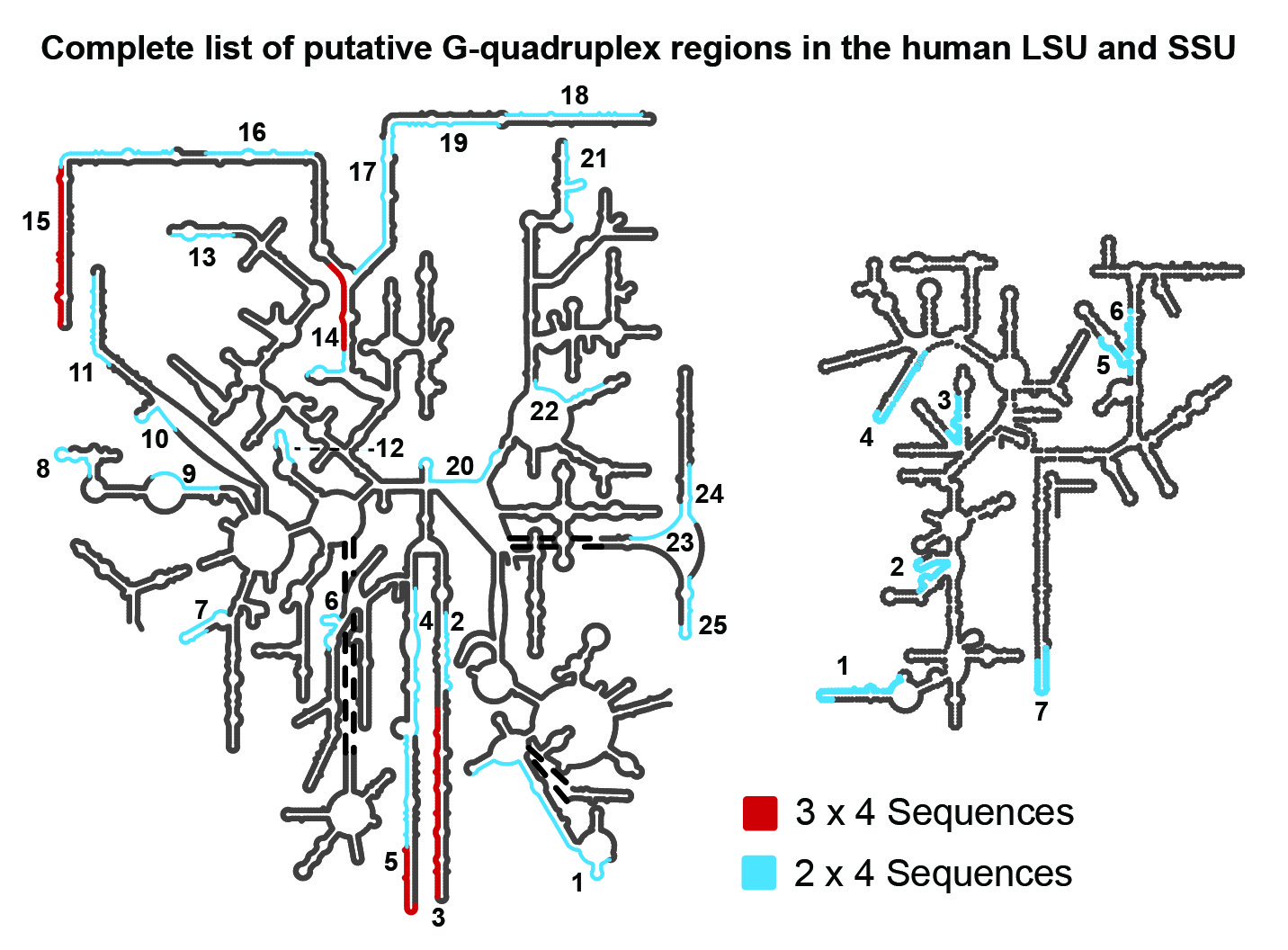
**

**Fig S2.** Secondary structures of the human LSU and SSU rRNAs with all identified 3 x 4 (red) and 2 x 4 (blue) G-quadruplex regions.

**
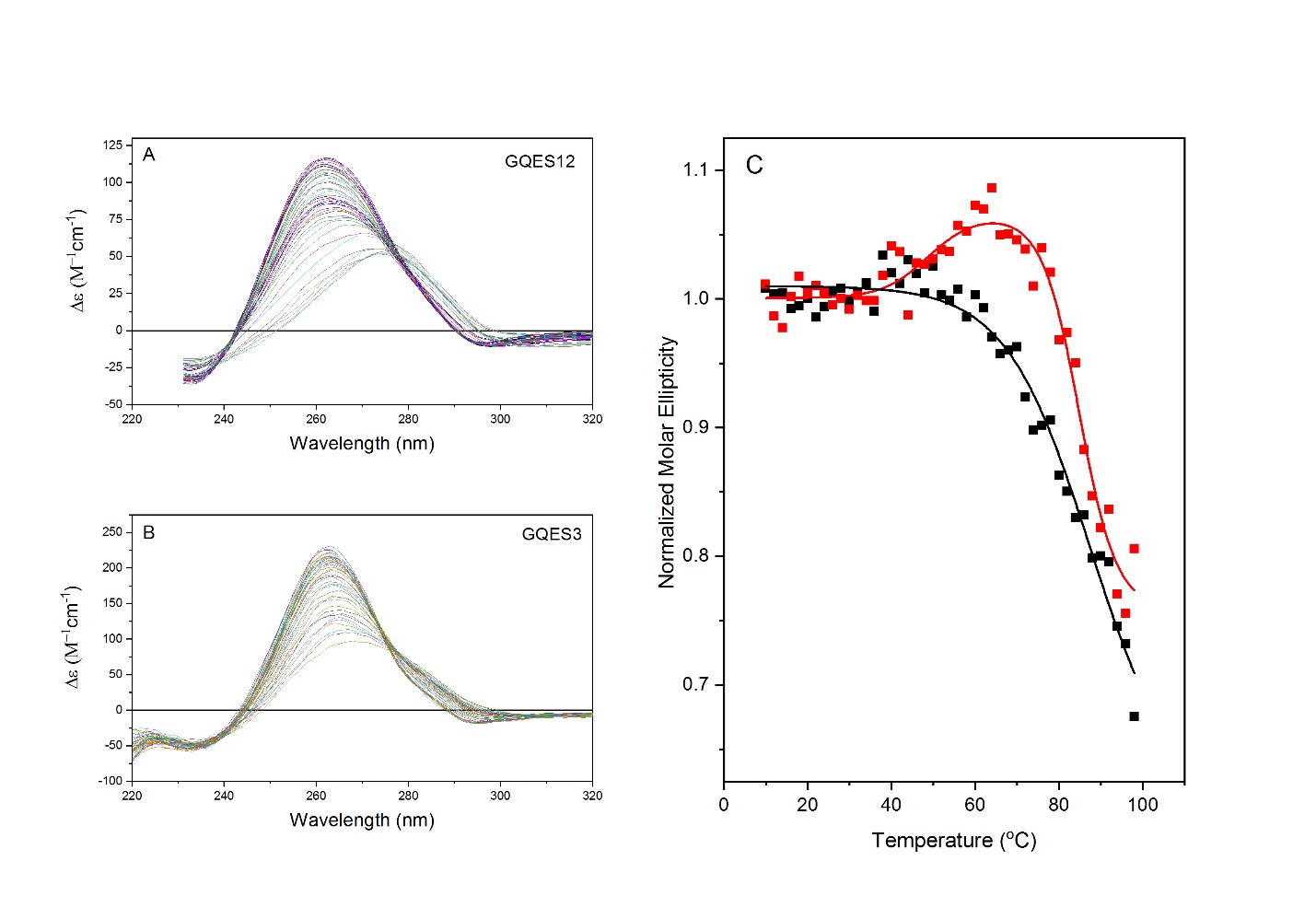
Fig S3.** Thermal denaturation of GQes12 (A) and GQes3 (B) as monitored by changes in circular dichroism. Panels A and B show the decrease in amplitudes in CD spectra as temperature is increased.

**
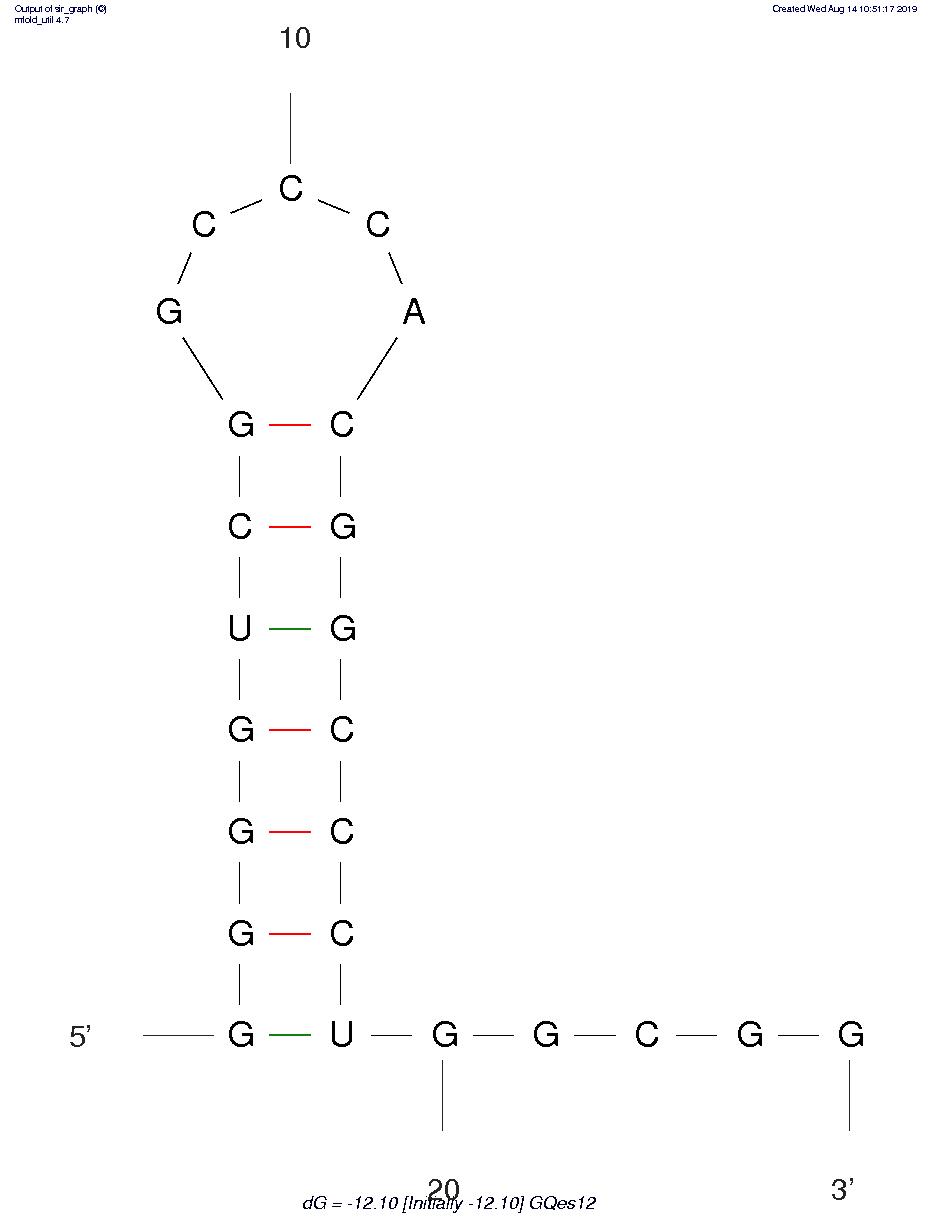
**

**Fig S4.** Possible hairpin structure of GQes12 calculated using the mFold RNA server.

**
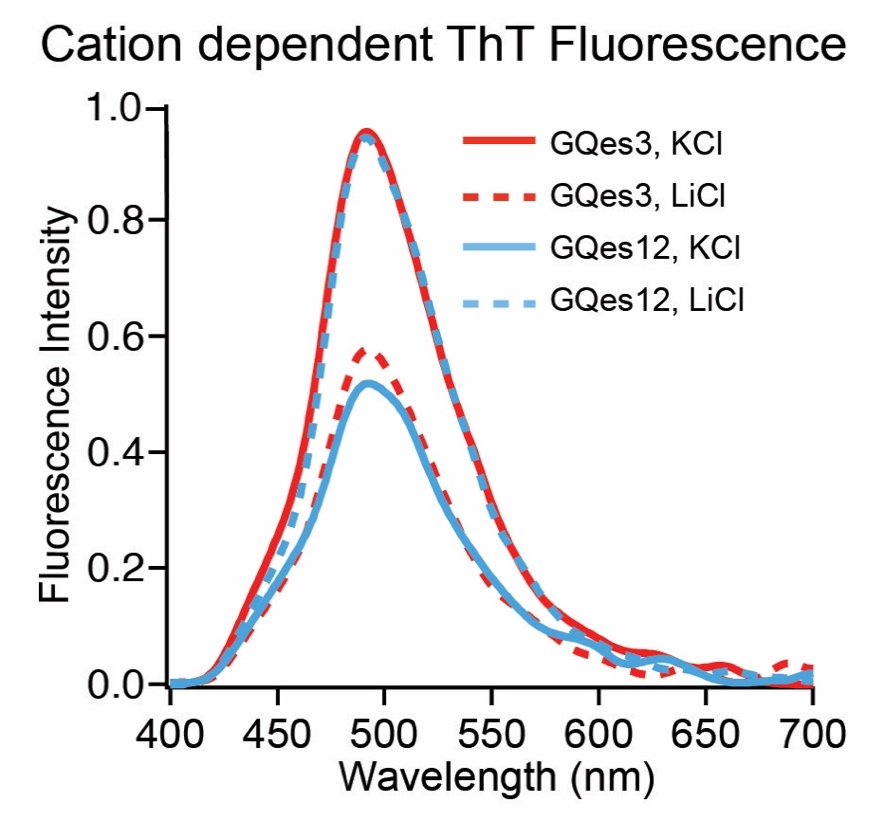
Fig S5.** Relative ThT fluorescence spectra of the GQes3 and GQes12 rRNA oligomers annealed in the presence of potassium or lithium ions.


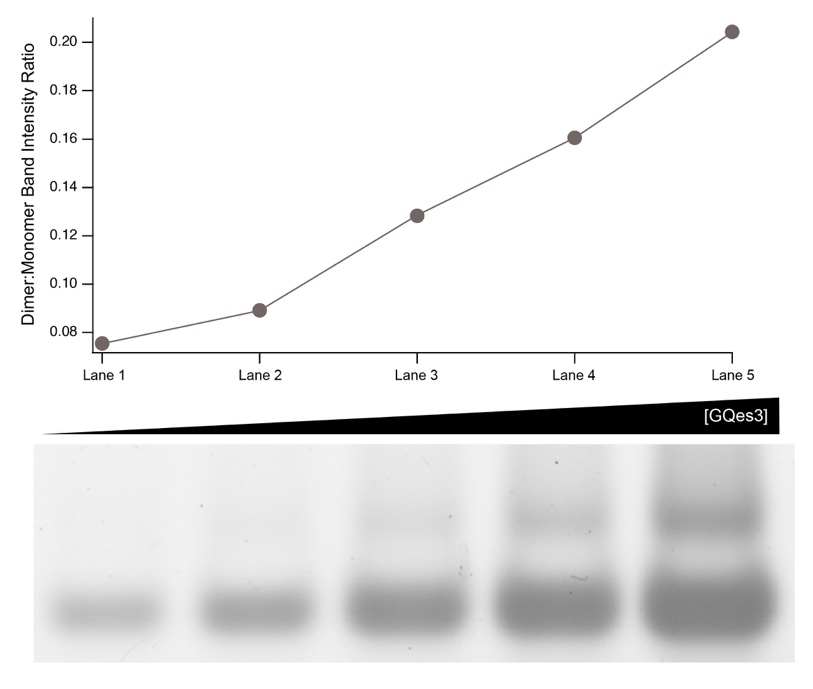


**Fig S6.** Increasing concentrations of GQes3 result in a shift from the monomer to the dimer species. Band intensities were quantified using ImageJ and the ratio of dimer to monomer was plotted. The increase in the ratio indicates that the monomer-dimer equilibrium is shifted to the latter upon the increase in the RNA concentration. RNA was resolved on a 6% Native PAGE.

**Table S1.** DNA and RNA sequences encoding RNAs used.

| Gene | Sequence (5’ to 3’) |
| --- | --- |
| GQES7-a | GAATTC*TAATACGACTCACTATAGGG*CGGA**GGGGG**C**GGG**CTCCGGC**GGG**TGC**GGGGG**T**GGG**C**GGG**C**GGGG**CC**GGGGG**T**GGGG**TCGGC**GGGGG**ACCGAAGCTT |
| GQES7-b | GAATTC*TAATACGACTCACTATAGGG*CCTC**GGG**A**GGG**CGCGC**GGG**TC**GGGG**CGGCAAGCTT |
| GQes3 | **GG**CCCC**GG**CC**GGGGGG**C**GGG**CGCC**GG** |
| *mut*es3 | **AA**CCCC**AA**CC**GAAAAG**C**GAA**GCC**AA** |
| GQes12 | **GGGG**UC**GG**CCCAC**GG**CCCU**GG**C**GG** |
| *mut*es12 | **AAAG**UC**AA**CCCAC**AA**CCCU**AA**C**GG** |

- The T7 promoter region is in italics, restriction sites are underscored (EcoRI 5’, HindIII 3’). G-tracts and G-tract mutations are underscored and in bold.
