# Supplementary material for: Profusion of G-quadruplexes on both subunits of metazoan ribosomes
Source: PLoS One. 2019 Dec 13;14(12):e0226177. doi: 10.1371/journal.pone.0226177 (PMC6910669; doi:10.1371/journal.pone.0226177)
Supplement: S1 Raw Images — (PDF) [file pone.0226177.s008.pdf]

**The following correspond to raw images obtained from gels in Fig. 3.**

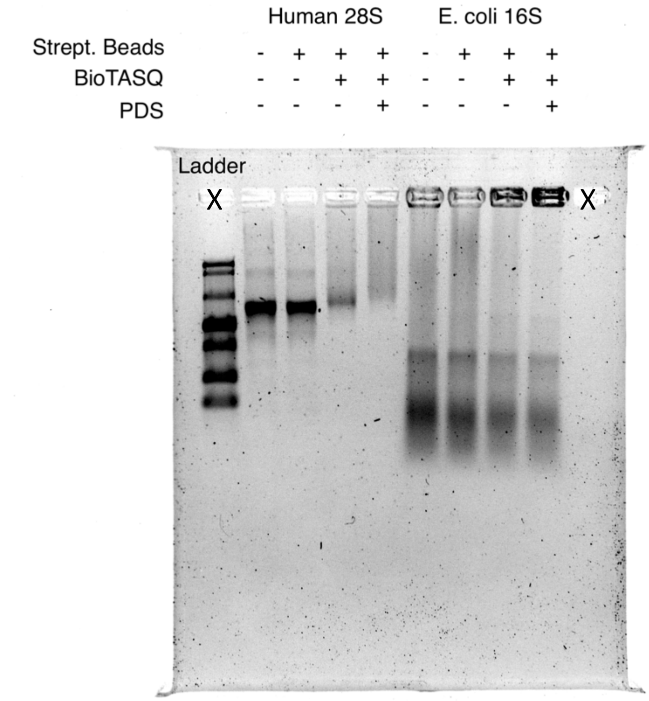


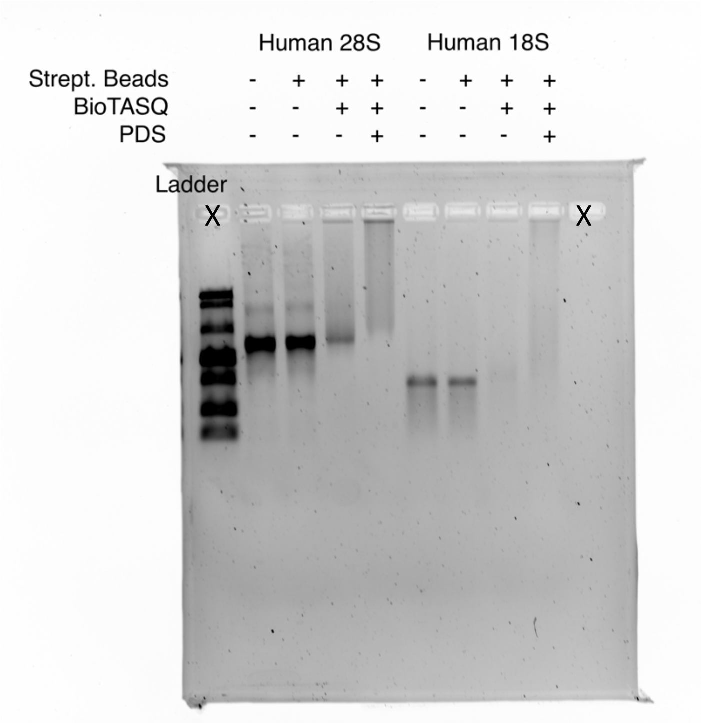


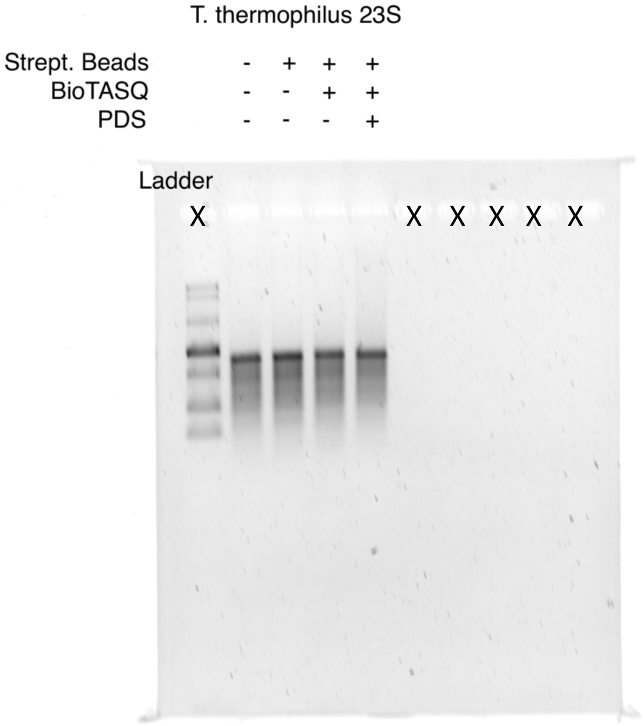


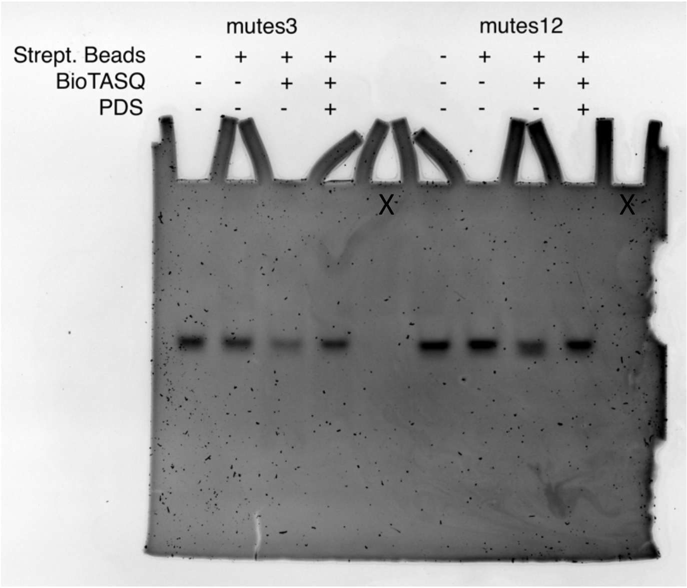

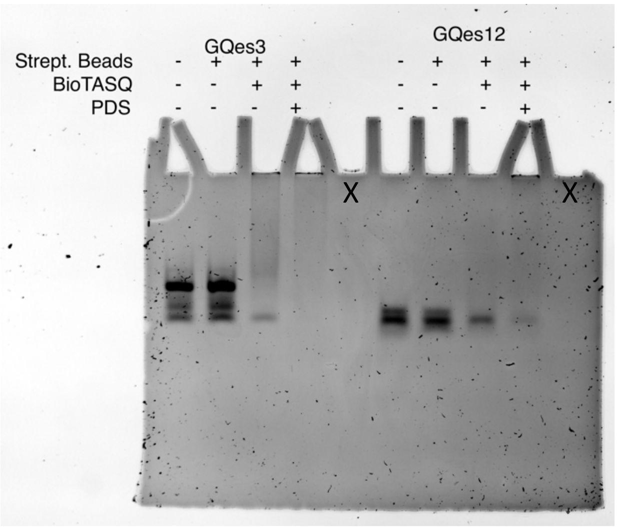


**The following correspond to the raw image obtained from gel in Fig. S.6**

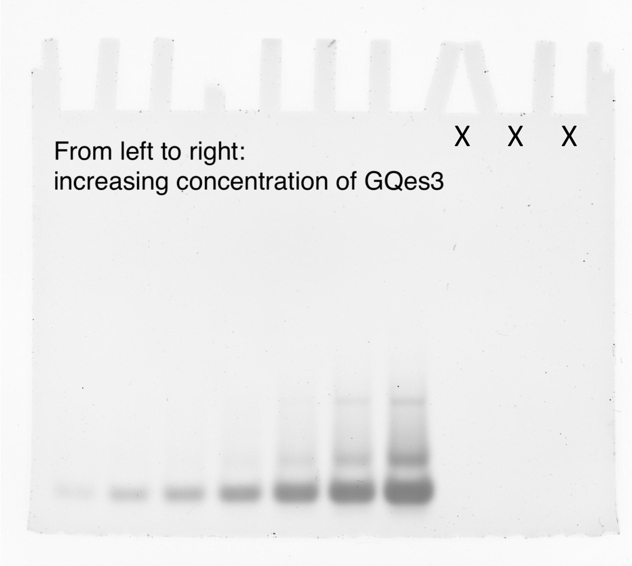


All images were taken with Azure Imager c400 (Azure Biosystems). An X above gel lanes is used to indicate lanes that were not used in the final cropped image that appears in the article.
